# Vitamin D

**DOI:** 10.1155/2010/631052

**Published:** 2010-03-03

**Authors:** Vin Tangpricha, Suzanne E. Judd, Diane Kamen, Yan Chun Li, Alan Malabanan

**Affiliations:** ^1^Division of Endocrinology, Metabolism and Lipids, Department of Medicine, Emory University School of Medicine, Atlanta, GA 30322, USA; ^2^Department of Biostatistics, University of Alabama at Birmingham, Birmingham, AL 35294, USA; ^3^Division of Rheumatology and Immunology, Medical University of South Carolina, Charleston, SC 29425, USA; ^4^Section of Gastroenterology, University of Chicago, Chicago, IL 60637, USA; ^5^Division of Endocrinology, Diabetes, & Metabolism, Harvard University, Boston, MA 02215, USA

The story of vitamin D continues to unfold almost 40 years after its discovery as a steroid hormone. The renaissance of vitamin D research began in the early 1990s when it became apparent that other tissues could produce the steroidal form of vitamin D locally and that the steroid form of vitamin D could regulate many functions important to several cellular processes. These initial observations were found in cells of the immune system followed by cancer cells. It is now known that vitamin D regulates over 900 genes and is involved in nearly every organ system in the human body. The potential that vitamin D has to modulate several organ systems coupled with early epidemiologic associations of low vitamin D status and several chronic disease states has resulted in a large newly formed scientific community focused on the investigation of vitamin D from the bench to bedside.

The manuscripts submitted to our special issue on vitamin D in the International Journal of Endocrinology demonstrate the wide breath of interest in vitamin D research. The vitamin D deficiency epidemic continues to be present internationally as reported by several of our submitted manuscripts. Classically, vitamin D deficiency results in inadequate mineralization of bone leading to osteoporosis; however, as reported by Straube and colleagues, vitamin D deficiency can also cause chronic pain which is often overlooked in clinical practice.

There continues to be interest in the effects of vitamin D on infancy and early adolescence and in populations with malabsorption. Several of our submitted manuscripts call attention to vitamin D deficiency in these populations. Wagner and colleagues provide evidence that early intervention with vitamin D in breast fed children can prevent vitamin D deficiency. Several groups have focused attention to the role of vitamin D in the cardiovascular system. In particular, our special issue has several manuscripts examining the role of vitamin D on lipid metabolism and glucose metabolism.

The story of vitamin D continues. Several chapters remain to be written. We still do not fully understand why the vitamin D hormone regulates so many different processes in the human. We remain optimistic that correction of vitamin D deficiency will reverse many of the epidemiologic associations of vitamin D deficiency with chronic medical illness.

*Vin Tangpricha* *Vin Tangpricha* *Suzanne E. Judd* *Suzanne E. Judd* *Diane Kamen* *Diane Kamen* *Yan Chun Li* *Yan Chun Li* *Alan Malabanan* *Alan Malabanan*

